# In Vitro Antiviral Activity of the Fungal Metabolite 6-Pentyl-α-Pyrone Against Bovine Coronavirus: A Translational Study to SARS-CoV-2

**DOI:** 10.3390/vetsci12070634

**Published:** 2025-07-02

**Authors:** Violetta Iris Vasinioti, Amienwanlen Eugene Odigie, Maria Stella Lucente, Luca Del Sorbo, Cristiana Catella, Elisabetta Casalino, Maria Michela Salvatore, Alessia Staropoli, Francesco Vinale, Maria Tempesta, Filomena Fiorito, Anna Andolfi, Alessio Buonavoglia, Annamaria Pratelli, Paolo Capozza

**Affiliations:** 1 Department of Veterinary Medicine, University of Bari, 70010 Valenzano, Italy; amienwanlen.odigie@uniba.it (A.E.O.); mariastella.lucente@uniba.it (M.S.L.); cristiana.catella@uniba.it (C.C.); elisabetta.casalino@uniba.it (E.C.); maria.tempesta@uniba.it (M.T.); annamaria.pratelli@uniba.it (A.P.); paolo.capozza@uniba.it (P.C.); 2 Department of Veterinary Medicine and Animal Production, University of Naples Federico II, 80137 Naples, Italy; luca.delsorbo2@studenti.unina.it (L.D.S.); mariamichela.salvatore@unina.it (M.M.S.); francesco.vinale@unina.it (F.V.); filomena.fiorito@unina.it (F.F.); 3 Department of Agricultural Sciences, University of Naples Federico II, 80055 Portici, Italy; alessia.staropoli@unina.it; 4 Institute for Sustainable Plant Protection, National Research Council, 80055 Portici, Italy; 5 Department of Chemical Science, University of Naples Federico II, 80126 Naples, Italy; anna.andolfi@unina.it; 6 Dental School, Department of Biomedical and Neuromotor Sciences, University of Bologna, 40126 Bologna, Italy; alessio.buonavoglia85@gmail.com

**Keywords:** antiviral activity, BCoV, 6-pentyl-α-pyrone, COVID-19

## Abstract

The recent COVID-19 pandemic has prompted the scientific community to prioritize the discovery of preventive methods and new therapeutics, including the investigation of natural compounds with antiviral potential. Fungal secondary metabolites (SMs) represent a promising source of antiviral drugs due to their structural diversity and intrinsic biocompatibility. Herein, the antiviral activity of 6-pentyl-α-pyrone (6PP) against bovine coronavirus (BCoV) has been evaluated in vitro. Considering that BCoV and SARS-CoV-2 are both members of the *Betacoronavirus* genus and share several key features, BCoV represents a valuable reference model for human coronavirus research. A non-cytotoxic dose of 6PP was used on MDBK cells to evaluate its antiviral activity against BCoV. Different experimental conditions were employed to examine cell monolayer protection both pre- and post-infection, as well as the potential inhibition of viral internalization. Overall, post-infection 6PP treatment reduced viral load and decreased viral internalization. Results were analyzed using viral titration and quantitative PCR, while data interpretation was performed by statistical software tools. This study presents a novel fluorescence quantification approach with high confidence demonstrated by its significant concordance with RT-qPCR results. These data suggest that 6PP could be an effective antiviral agent for BCoV, warranting further investigation of its role in coronavirus inhibition.

## Simple Summary

This study aimed to evaluate the activity of the fungal metabolite 6-pentyl-α-pyrone against bovine coronavirus in vitro. Recently, the COVID-19 crisis has highlighted the urgent need to investigate natural products and their derivatives as potential antiviral drugs. Moreover, bovine coronavirus is closely related to human coronaviruses, and it is often used as a virus model in preliminary antiviral research to avoid handling highly pathogenic human viruses. Herein, it was demonstrated that 6-pentyl-α-pyrone presents antiviral activity against bovine coronavirus under specific conditions, and further research is recommended to clarify its potential in coronavirus therapeutics.

## 1. Introduction

Bovine coronavirus (BCoV) is a positive single-stranded RNA virus within the genus *Betacoronavirus* in the *Coronaviridae* family. The genus *Betacoronavirus* includes, among others, human coronavirus (HCoV) OC43 and viruses associated with the two recent epidemics, Severe Acute Respiratory Syndrome Coronavirus 1 (SARS-CoV-1) and Middle East Respiratory Syndrome-Coronavirus (MERS), as well as the pandemic SARS-CoV-2 [1,2].

BCoV circulates in cattle worldwide, causing respiratory and enteric disease, but can also be detected in asymptomatic animals [3,4]. The virus is associated with three distinct clinical syndromes, namely, calf diarrhea, winter dysentery (WD) with hemorrhagic diarrhea in adults, and bovine respiratory disease complex (BRDC) or shipping fever of feedlot cattle [5,6,7,8]. BCoV infection is characterized by high morbidity and mortality rates, resulting in significant losses in the livestock industry [9,10]. Although enteric and respiratory BCoV strains exhibit genetic differences at the spike gene level, only a single BCoV serotype has been identified to date, capable of conferring cross-protection among isolates [11]. Therefore, enteric and respiratory isolates belong to the same quasispecies with variations in clinical signs likely resulting from interactions among the virus, host, and environment [7,12].

As members of the *Betacoronavirus* genus, BCoV and SARS-CoV-2 share some common pathogenic features, and several studies have proposed the use of BCoV as a virus model to study SARS-CoV-2 [3]. Sequence homology analysis of the aminoacidic sequence of spike and nucleocapsid proteins of BCoV and SARS-CoV-2 showed only 38.4% and 38.9% identity, respectively [13,14]. However, despite the low overall amino acid similarity, comparative analysis of the major antigenic epitopes of these proteins revealed a significantly higher degree of homology [15].

Recent advancements in the pharmaceutical industry have highlighted the potential of natural compounds as antiviral agents [16,17], with nearly 80% of commercially available drugs derived from natural sources [18]. Fungal secondary metabolites (SMs), in particular, are appreciated for their unique chemical structures and their diverse biological properties, including low molecular weight, structural diversity, and bioactivity [19,20,21,22,23]. Moreover, the industrial production of fungi-derived metabolites offers a cost-effective alternative to synthetic drug development [24]. Recently, several studies have evaluated the antiviral activity of fungal SMs against animal and human viruses [25,26,27,28]. Among these, alpha-pyrones have been reported to exhibit protease inhibition activity [29] and have been suggested as potential inhibitors of CoVs [30]. The fungal metabolite 6-pentyl-α-pyrone (6PP) has been recently reported to reduce canine coronavirus infection, inducing a significant downregulation in the expression level of the viral nucleocapsid protein [25]. To date, there is no other literature available regarding 6PP’s antiviral efficacy against other viruses or in other animal species.

In the present study, the antiviral activity of the 6PP against BCoV was evaluated in vitro. The aim was to promote the research and development of antivirals for the treatment of SARS-CoV-2 infection, using an “in vitro” model that allows preclinical studies aimed to evaluate the efficacy of antiviral drugs, bypassing the difficulties and the risks deriving from the use of a highly pathogenic and contagious virus for humans in the first phases of screening. In addition, this study presents a novel and alternative strategy for the precise quantification of fluorescence, consistent with RT-qPCR results, with several potential research applications.

## 2. Materials and Methods

### 2.1. Production and Isolation of 6PP

6-Pentyl-α-pyrone (6PP) was isolated from *Trichoderma atroviride* strain P1 according to the method described by Vinale et al. [31]. Briefly, five 10 mm Ø plugs, obtained from actively growing P1 cultures, were inoculated into 5 L conical flasks containing 2.5 L of potato dextrose broth (PDB, HI-MEDIA, Pvt. Ltd., Mumbai, India). Two static cultures were incubated for 30 days at 25 °C and then filtered under vacuum through Miracloth filter paper (Sigma-Aldrich, St. Louis, MO, USA).

Culture filtrates (5 L) were exhaustively extracted with ethyl acetate (EtOAc, VWR International, LLC, Milan, Italy), the organic phase was dried with sodium sulfate anhydrous (Na_2_SO_4_, VWR International) and evaporated under vacuum at 37 °C. Purification of 6PP was achieved by flash column chromatography (silica gel as stationary phase, 100 g) with an elution gradient composed of petroleum ether (Carlo Erba, Milan, Italy) and EtOAc (0–100% EtOAc). Characterization of purified 6PP was achieved by gas chromatography-mass spectrometry (GC-MS) analysis according to the method reported by Staropoli et al. [32].

### 2.2. Cell Culture and Virus

Madin Darby bovine kidney (MDBK) cells were maintained at 37 °C in a humidified atmosphere containing 5% CO_2_ using Dulbecco’s Modified Essential Medium (DMEM) supplemented with 10% fetal bovine serum, 100 IU/mL penicillin, 0.1 mg/mL streptomycin, and 2 mM L-glutamine. The same culture medium was utilized for the antiviral assays.

The BCoV strain 438/06, isolated from a 2–3-month-old cattle, was propagated and titrated in MDBK cells. The resulting viral stock, exhibiting a titer of 10^−7^ Tissue Culture Infectious Dose per 50 μL (TCID_50_/50 μL), was aliquoted and stored at −80 °C for use in the subsequent experimental procedures.

### 2.3. Viral Titration

Serial ten-fold dilutions (ranging up to 10^−8^) of each collected supernatant were titrated in quadruplicate in 24-well plates containing MDBK cells. The plates were incubated for 72 h at 37 °C in a 5% CO_2_ atmosphere, and then, the cells were tested with the Indirect Immunofluorescence (IF) test using a specific bovine serum positive to BCoV and the anti-bovine IgG fluorescein conjugated serum (Sigma Chemicals, St. Louis, MO, USA). The titer was expressed as the highest virus dilution showing fluorescent foci.

### 2.4. Cytotoxicity Assay

The cytotoxicity of the 6PP was determined using the in vitro Cell Proliferation Kit (Sigma–Aldrich Srl, Milan, Italy), based on 3-(4,5-dimethylthiazol-2-yl)-2,5-diphenyltetrazolium bromide (MTT). The toxicity of the compound was measured on confluent monolayers of MDBK cells in 96-well microtiter plates. Trypan Blue exclusion test, as previously described [33], was employed to assess cell viability, indicating a non-cytotoxic 6PP concentration of 0.1 μg/mL. Based on these results, two-fold serial dilutions of the 6PP, from 0.25 μg/mL to 0.002 μg/mL, were performed to encompass the non-cytotoxic concentration within the range of tested values. The diluted compound was added to the 96-well plates and incubated at 37 °C for 24 h in a 5% CO_2_ incubator. After incubation, 10 μL of MTT labeling reagent (0.5 mg/mL) was added to each well for 4 h at 37 °C, and, subsequently, 100 μL of solubilization buffer was added to solubilize the formazan crystals. The following day, the optical density (OD) was measured by an automatic spectrophotometer (iMark™ Microplate Absorbance Reader) at a wavelength of 570 nm (with reference wavelength = 655 nm). Each experiment included a control consisting of complete medium and untreated cells. 6PP cytotoxicity percentage was calculated according to the following formula: % Cytotoxicity = [(OD of control cells−OD of treated cells) ×100]/OD of control cells.

The maximum non-cytotoxic concentration was defined as the concentration at which viability of the treated MDBK cells was reduced by 20% compared to untreated control cells (IC20). All experiments were conducted in quadruplicate to ensure reproducibility and statistical reliability.

### 2.5. TBARS Assay

Thiobarbituric acid-reactive substance (TBARS) levels were determined on the supernatant of untreated MDBK cells after 24 h incubation and on the supernatant of cells treated with 6PP at different concentrations (0.25, 0.125, 0.0625 μg/mL), using a modified version of the Buege and Aust method [34]. Briefly, 100 mL of culture medium was mixed with 200 mL of trichloroacetic acid (TCA) to eliminate any proteins present in the medium. After centrifugation, 200 mL of the supernatant was added to 200 mL of 0.67% thiobarbituric acid (TBA) solution and incubated at 90 °C for 15 min. At the end of incubation, 150 mL of solution was placed into a 96-well plate, and absorbance was measured at a wavelength of 532 nm. TBARS concentration was expressed as micromoles/L using 1,1,3,3-tetramethoxy-propane as a standard.

### 2.6. Viricidal Activity Assay

The potential viricidal activity of 6PP against BCoV was evaluated. The IC20 concentration was mixed with an equivalent volume of 100 TCID_50_/50 μL of BCoV and incubated for different times (10, 30, and 60 min) at 4 °C, 37 °C, and room temperature (RT). In parallel, for each assay, a mixture of TCID_50_/50 μL of BCoV and DMEM (control virus) was prepared. Both the virus–compound and virus–DMEM mixtures were distributed into different wells of cell monolayers cultured in 24-well plates. Ten-fold serial dilutions were performed to assess the viral titers of each mixture. The plates were incubated at 37 °C for 1 h to allow virus adsorption. Then, the inoculum was carefully replaced with DMEM and the plates were incubated for 72 h at 37 °C in a CO_2_ incubator.

### 2.7. Antiviral Activity Assays

Based on the results of the cytotoxicity assay, the antiviral activity against BCoV was evaluated using 6PP at the established IC20 dose. To assess the specific phase of viral replication compromised/inhibited by the 6PP, three distinct experimental protocols (A, B, and C) were applied. The experiments were conducted in triplicate.

#### 2.7.1. Protocol A: Cell Protection After Viral Infection

MDBK cells were seeded in 24-well plates, and after 24 h, the cells were infected with 100 μL of BCoV containing 100 TCID_50_/50 μL. After virus adsorption for 1 h at 37 °C, the inoculum was removed, and the cell monolayers were washed with DMEM. Two distinct experimental conditions were then adopted:

(1) The IC20 concentration of the 6PP was added to the cell monolayers, and plates were incubated for 72 h at 37 °C.
(2) The IC20 concentration of the 6PP was added to the cell monolayers, and plates were incubated for 3 h at 37 °C. Following incubation, the compound was removed, the wells were washed with DMEM, and then, replaced with 1 mL of DMEM, and the plates were further incubated for 72 h at 37 °C.

In untreated infected cells (control virus), DMEM was used to replace the inoculum.

Three days post-infection, aliquots of the supernatants were collected, and virus titration and RNA quantification were performed.

#### 2.7.2. Protocol B: Cell Protection Before Viral Infection

MDBK cells were seeded in 24-well plates, and after 24 h, cell monolayers were incubated with the IC20 concentration of 6PP (1 mL) for 3 h under two distinct experimental conditions: 4 °C and 37 °C. After the incubation time, 6PP was removed from each well, and all the monolayers were infected with 100 μL of BCoV containing 100 TCID_50_/50 μL for 1 h at 37 °C. After virus adsorption, the inocula were replaced with DMEM.

In untreated infected cells, DMEM was employed to replace the inocula (control virus). After 72 h incubation, aliquots of the supernatants were collected, and virus titration and RNA quantification were performed.

#### 2.7.3. Protocol C: Viral Internalization Inhibition Assay

MDBK cells were seeded in 24-well plates, and after 24 h, infected with 100 μL of BCoV containing 100 TCID_50_/50 μL. Cells were then incubated for 1 h at 4 °C, allowing virus adsorption but not internalization. After incubation, the IC20 concentration of 6PP was added to each well.

In untreated infected cells, DMEM was used to replace the inocula (control virus). After 72 h, aliquots of the supernatants were collected, and virus titration and RNA quantification were performed.

### 2.8. Fluorescence Quantification

Fluorescence image quantification was performed using Python version 3.9.18 (https://docs.python.org/3/reference/index.html; Accessed 3 March 2025) with the initiation of several core libraries and their respective dependencies in the Python programming environment. These included Open-Source Computer Vision Library (OpenCV) with capabilities for image processing, Pandas for high-performance data analysis, Numerical Python (NumPy) for array operations, and Matplotlib version 3.5.2 and Seaborn version 0.11.2 for interactive and statistical data visualizations, as well as Scikit-learn and SciPy for data preprocessing and evaluation [35,36,37,38]. Briefly, experimental and control images were grayscale-transformed to two-dimensional NumPy arrays with the inverse binary thresholding method. Congruence was achieved between the positive and negative fluorescence images using an iterative optimization function that refined image value parameters and facilitated optimum threshold selection. Next, an HSV (Hue, Saturation, and Value) color space transformation into a three-channel representation of color attributes across rows, columns, and color components enabled bounded adjustment and parametrization over the blue and green hue channels. Finally, for each identified vectorized fluorescent image, pixel intensity values were aggregated by calculating the mean across all pixels within the image. The complete code implementation and plot visualizations are provided in the Supplementary Materials (HTML file and Figure S1).

### 2.9. RT-qPCR for BCoV

Total RNA was isolated from 200 μL of each supernatant using the IndiSpin^®^ Pathogen Kit (Indical Bioscience GmbH, Leipzig, Germany) following the manufacturer’s guidelines. The purified RNA was promptly stored at −80 °C until further processing by RT-qPCR analysis. Complementary DNA (cDNA) synthesis was conducted in a 10 μL reaction volume employing random hexamers and MuLV reverse transcriptase (GeneAmp^®^ RNA PCR, Applied Biosystems, Applera Italia, Monza, Italy), adhering strictly to the protocol provided by the manufacturer for BCoV detection.

Subsequently, the RT-qPCR was performed utilizing previously described primers and TaqMan probe, along with identical cycling parameters and reagent concentrations [6]. Specifically, 10 μL of synthesized cDNA was combined with 15 μL of reaction mixture containing IQ™ Supermix (Bio-Rad Laboratories Srl, Segrate, Italy), 0.6μM of each primer, and 0.4 μM of probe. The thermal cycling protocol after a polymerase (iTaq DNA polymerase) activation step at 95 °C for 10 min, comprised 45 amplification cycles consisting of denaturation at 95 °C for 10 s and combined annealing extension at 56 °C for 30 s. Amplification and data acquisition were carried out using the i-Cycler iQTM Real-Time Detection System (Bio-Rad Laboratories Srl), and the resulting data were analyzed using the corresponding software (version 3.0).

### 2.10. Data Analysis

The OD absorbance values obtained were converted into percentages, and the cytotoxicity results of 6PP were analyzed using non-linear curve fitting. Moreover, a dose–response curve was elaborated through non-linear regression analysis to evaluate goodness of fit (GraphPad Prism 10.3.1 program, Intuitive Software for Science, San Diego, CA, USA). From the fitted dose–response curve, IC20 was assessed. The results were validated using Python (version 3.9.18), ensuring both accuracy and reproducibility.

Statistical analysis was performed in the Python programming environment as described previously. Analyses of viricidal activities of active metabolite were performed using repeated-measures ANOVA (rm-ANOVA), considered the most appropriate for data analysis given the repeated measures design, the correlated nature of the data, and the presence of multiple factors of time, temperature, and dilution. In addition to the uncorrected *P*-value (*P*-unc), the analysis also reported the Greenhouse–Geisser correction (*P*-corr) to address the potential violation of the sphericity assumption potentially inherent in repeated-measures designs, thus enhancing robustness of the statistical inferences. Where a statistical significance was found, a pairwise post hoc comparison was performed using the Pingouin (pg) library [39,40]. Pingouin is a user-friendly Python library specifically designed for common statistical tests and has a pairwise function call specifically designed for multiple comparisons following a significant rm-ANOVA. The Bonferroni correction was applied to adjust for multiple comparisons, which helps maintain a stringent level of significance and ensure that the observed differences are truly meaningful. The results are presented in the table and figures below.

In contrast, the computed mean fluorescence, serving as the continuous dependent variable, was subjected to a two-way analysis of variance (ANOVA) to comprehensively determine the significance of the various categorical factors across all experimental conditions. This analysis was conducted within the statsmodels library with its established Ordinary Least Squares (OLS) functionality for robust model estimation.

## 3. Results

### 3.1. Cytotoxicity Assay

**Figure 1:**
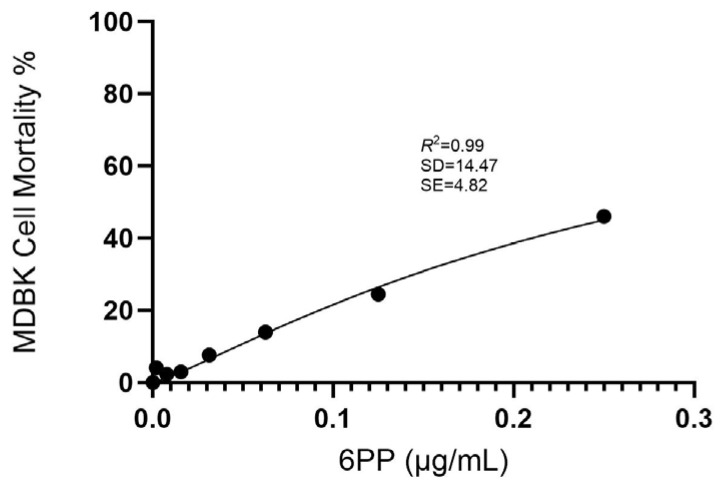
Dose–response curve for 6PP generated using GraphPad Prism 10.3.1 program, Intuitive Software for Science, San Diego, CA, USA. The *x*-axis represents the concentration of the compound (µg/mL), while the *y*-axis shows the percentage of cell mortality (%). The curve was fitted using a nonlinear regression analysis. Results were validated in Python Programming Language 3.9.18, with calculations of R^2^ (coefficient of determination), SD (Standard Deviation), and SE (Standard Error). R^2^ indicates the proportion of observed variance in the independent variable that is explained by the independent variables within the regression model.

The cytotoxicity of 6PP was determined by measurement of cell viability with the MTT colorimetric method after exposing the cells to various concentrations. Cytotoxicity was assessed by measuring the absorbance signal spectrophotometrically. Based on the adjusted dose–response curve, the IC20 value of 6PP was calculated to be 0.1 μg/mL (Figure 1).

### 3.2. TBARS

Oxidative stress was evaluated by measuring TBARS production. The results indicate that 6PP did not induce oxidative stress in MDBK cells in any of the tested concentrations, and the TBARS levels were lower compared to those in the supernatant of untreated MDBK cells (1.95 μM).

### 3.3. Virucidal Activity

**Figure 2:**
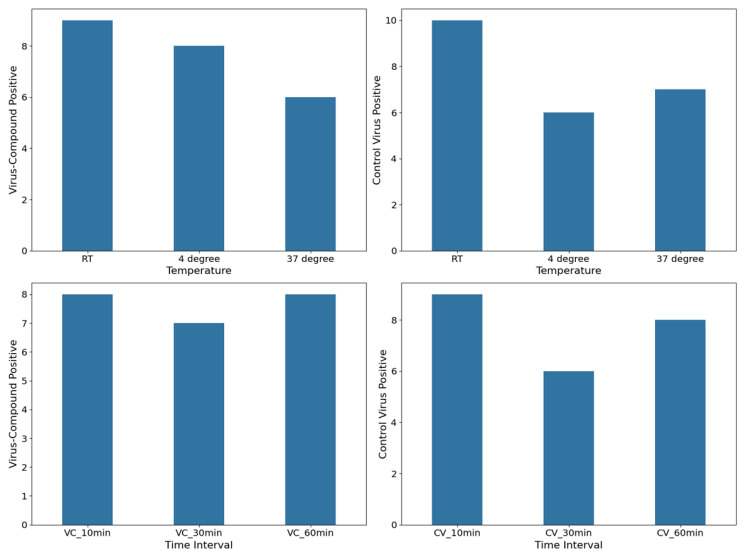
Positive immunofluorescence obtained for both the virus–compound (VC) mixture group and the control virus (CV) mixture group at different time intervals and room temperature (RT).

The results of samples that yielded positive immunofluorescence (positive samples) obtained for both the virus–compound mixture group and the control virus mixture group are shown in Figure 2, where the frequency of observed positives at various point measurements for temperature and various time intervals for virus–compound and control virus, respectively, is reported. The numbers of positive samples in the virus–compound mixture group were relatively consistent across temperatures, with nine at room temperature (RT), eight at 4 °C, and six at 37 °C. Similarly, the control virus mixture group exhibited a comparable trend, with ten positive samples at RT, followed by six at 4 °C and seven at 37 °C. The virus–compound mixture exhibited a peak in positive results at 10- and 60 min post-treatment with eight positives each, while the control virus group exhibited similar trends with nine, eight, and six positive results recorded at 10, 30, and 60 min, respectively.

**Figure 3:**
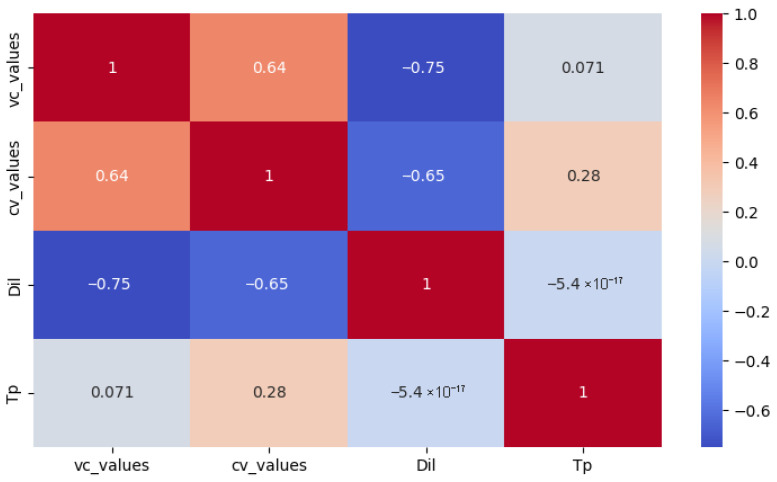
Correlation analysis between virus–compound (vc_values)/control virus values (cv_values) and the various temperatures (Tp) and dilutions (Dil).

A moderately high negative correlation was found between dilution and the total number of positive samples in the immunofluorescence test for both virus–compound mixture (R = −0.75) and control virus (R = −0.65) (Figure 3).

The rm-ANOVA analysis revealed no statistically significant differences at any of the tested time intervals between the virus–compound mixture treatment group and the control virus treatment group, across the various dilutions (*p* = 0.775) and temperatures (*p* = 0.192). A similar observation was found for within variables of each treatment group (Table S1).

Post hoc analyses were used to identify the significant pairwise differences, both with and without Bonferroni multiple testing correction. Pairwise comparisons revealed significant differences using the standard alpha level, but these differences were not sustained under the more conservative Bonferroni correction (Table S2).

### 3.4. Antiviral Activity

The results of the antiviral activity test were assessed by viral cDNA quantification and IF assays, conducted 72 h after completing each antiviral protocol (A, B, and C). The supernatants collected from the different antiviral assays were tested with a BCoV-specific RT-qPCR protocol. The cDNA viral load of untreated infected cells was used as a reference and was calculated as the mean of the values obtained from each protocol (mean = 5.9 log10 cDNA viral copy numbers).

The plates were examined using an inverted fluorescence microscope, while the images of the IF assay were acquired and subsequently analyzed using an in-house image analysis code to quantify fluorescence intensity and assess antiviral efficacy. The mean fluorescence of untreated infected cells was used as a reference and was calculated as the mean of the values obtained from the corresponding fluorescence images for each protocol (mean = 5.18 brightness intensity levels).

#### 3.4.1. Protocol A: Cell Protection After Viral Infection

(1) Treatment of cell monolayer with 6PP for 72 h: comparing the log10 viral cDNA copies/mL of 6PP-treated infected cells (mean = 3.85 log10 cDNA viral copy numbers) with untreated infected cells (mean = 5.9 log10 cDNA viral copy numbers), a significant decrease of 2.02 log10 (*p* = 0.0175) was detected in treated cells. Similarly, a statistically significant difference in mean fluorescence was observed between infected cells treated with 6PP for 72 h (mean = 0.04 brightness intensity levels) and untreated infected cells (mean = 5.18 brightness intensity levels) (*p* = 0.0169).
(2) Treatment of cell monolayer with 6PP for 3 h: comparing the log10 viral cDNA copies/mL of 6PP-treated infected cells (mean = 6.02 log10 cDNA viral copy numbers) with untreated infected cells (mean = 5.9 log10 cDNA viral copy numbers), a difference of 0.12 log10 was observed without any statistical significance (*p* = 0.4465). Similarly, the mean fluorescence of the cell monolayer treated with 6PP for 3 h (mean = 2.58 brightness intensity levels) and untreated infected cells (mean = 5.18 brightness intensity levels) was not significantly different (*p* = 0.127).

#### 3.4.2. Protocol B: Cell Protection Before Viral Infection

(1) Treatment with 6PP at 4 °C: the log10 viral cDNA copies/mL of 6PP-treated infected cells (mean = 6.16 log10 cDNA viral copy numbers) compared with the untreated infected cells (mean = 5.9 log10 cDNA viral copy numbers) revealed a 0.26 log10 difference, although without any statistical significance (*p* = 0.0924). This is consistent with the fluoroscopy quantification method, which did not reveal any statistical significance (*p* = 0.964) between the infected cells treated with 6PP at 4 °C versus untreated infected cells, with computed mean pixels of 5.26 and 5.18 brightness intensity levels, respectively.
(2) Treatment with 6PP at 37 °C: when comparing the log10 viral cDNA copies/mL of 6PP-treated infected cells (mean = 5.77 log10 cDNA viral copy numbers) with untreated infected cells (mean = 5.9 log10 cDNA viral copy numbers), no statistically significant difference was observed (*p* = 0.4223). Consistent with these results, the fluorescence quantification technique did not detect any significant difference (*p* = 0.147) in the mean fluorescence between infected cells treated with 6PP at 37 °C (mean = 2.59 brightness intensity levels) and untreated infected cells (mean = 5.18 brightness intensity levels).

#### 3.4.3. Protocol C: Viral Internalization Inhibition Assay

Comparing the log10 viral cDNA copies/mL of 6PP-treated infected cells (mean = 3.26 log10 cDNA viral copy numbers) to that of untreated infected cells (mean = 5.9 log10 cDNA viral copy numbers), a significant reduction of 2.64 log10 in viral load of treated cells was observed (*p* = 0.0003). In comparison, our quantification method detected a statistically significant difference in mean fluorescence between infected cells treated with 6PP (mean = 0.032 brightness intensity levels) compared with untreated (mean = 5.18 brightness intensity levels) infected cells (*p* = 0.0168).

**Figure 4:**
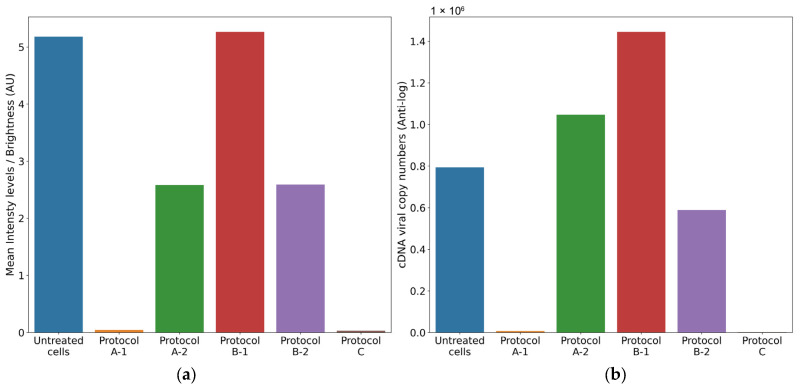
Fluorescence (**a**) and cDNA viral copies (**b**) quantification detected in the different antiviral protocols. AU = Arbitrary intensity Units, Protocol A-1: Treatment of cell monolayer with 6PP for 72 h, Protocol A-2: Treatment of cell monolayer with 6PP for 3 h, Protocol B-1: Treatment with 6PP at 4 °C, Protocol B-2: Treatment with 6PP at 37 °C, Protocol C: Viral internalization inhibition.

The comparison between the results obtained using the fluorescence quantification approach and the RT-qPCR is reported in Figure 4. To precisely illustrate the magnitude of the differences of the distinct outcomes observed between the various protocols, we present the actual cDNA copy numbers directly, rather than scaled logarithmic transformations that could obscure subtle but important differences. In this study, mean intensity levels were reported as arbitrary intensity units (AU) due to the absence of pre-calibration standardization necessary to map pixel intensities to absolute radiometric values. Future investigations with the availability of camera calibration data (e.g., exposure time and quantum efficiency) would enable results to be expressed in standardized units such as photons/seconds or μW/cm^2^.

## 4. Discussion

SARS-CoV-2 is a highly infectious pathogen; therefore, its use in experimental research is challenging and requires specialized personnel and laboratories. Several studies have proposed BCoV as an important reference virus for HCoV research due to the common features among β-coronaviruses and their high homology in the major antigenic epitopes of the spike and the nucleocapsid protein [3,15]. Consequently, in order to identify new effective molecules against SARS-CoV-2 while circumventing the use of a high-risk human pathogen, the aim of the present study was to use BCoV as a surrogate model for a preliminary antiviral efficacy evaluation test of 6PP.

The present study highlights the potential antiviral activity of 6PP, a fungal bioactive compound identified as a protease inhibitor, against BCoV in vitro. Beyond this crucial finding, to better determine the efficacy of 6PP in cell cultures infected with BCoV, a novel methodology for fluorescence quantification that shows remarkable concordance with consolidated RT-qPCR results was introduced. These results are highly significant and represent a direct and crucial translational pathway for rapid and reliable assessment of the antiviral efficacy of natural drugs in vitro, with immediate implications for the ongoing fight against SARS-CoV-2.

These studies involved the use of a non-cytotoxic dose of 6PP (0.1 μg/mL) in vitro on MDBK cells to evaluate its effects on different phases of BCoV infection. Several experimental conditions were employed to examine cell monolayer protection both pre- and post-infection, as well as the potential inhibition of viral internalization. A statistically significant reduction in BCoV load was observed when the cell monolayer was treated with the 6PP for 72 h post-infection. These results indicate that 6PP can interfere with the BCoV replication cycle, exhibiting antiviral potential. Moreover, the treatment with 6PP following inhibition of virus internalization also resulted in a significant decrease in viral load, implying that 6PP may limit the mechanisms of viral entry. These results are consistent with previous studies that have demonstrated the antiviral efficacy of 6PP on canine coronavirus (CCoV) [25] and suggest that 6PP exhibits antiviral activity both in the early and in the late stages of BCoV infection. Furthermore, another important finding was the reduction in TBARS production at all tested 6PP concentrations. This antioxidant effect may be due to the activation of antioxidant defenses, as reported by other authors in different cellular systems [41,42].

Importantly, this study introduces a novel methodology for precise fluorescence quantification, which also demonstrates remarkable concordance with results obtained via the established RT-qPCR technique. Critically, compared to the high cost of conventional RT-qPCR, this method offers a significantly cheaper, faster, and broadly applicable alternative, with greater resilience to variations in controlled environmental conditions. We posit that this adaptable method holds considerable potential for integration with other quantification approaches, as previously described [43]. While acknowledging potential limitations related to optimal parameterization and threshold selection, as well as the known influence of the acquired image field on the quantification accuracy, we suppose that further optimization through Bayesian modelling and the adoption of appropriate image-capturing instrumentation can effectively mitigate these sources of error. Indeed, a future implementation focused on rigorous parameter refinement promises to fully unleash the potential of this versatile and cost-effective fluorescence quantification method.

Another limit of this research is that the selection of BCoV as a model virus to study SARS-CoV-2 is largely based on the epitope-level similarities between the two viruses, despite relatively low amino acid similarity in the spike and nucleocapsid proteins. While these share epitopes may indeed induce “cross-reactive” immune responses, there are other significant genetic and biological differences that may be critical. Nevertheless, BCoV could be used as a pragmatic approach to better study some aspects of SARS-CoV-2, rather than an ideal surrogate. Moreover, the lack of in vivo validation for 6PP’s safety and efficacy is acknowledged as a limitation of this study. However, the bovine species represents the only currently available and reliable animal model for BCoV infection, and the implementation of in vivo trials in calves poses considerable ethical and financial challenges. Future in vivo testing may be more appropriately designed upon obtaining in vitro conclusive data and a better understanding of the exact mechanism of 6PP.

Other natural compounds, including flavonoids, plant extracts, and essential oils, have demonstrated potential antiviral activity against SARS-CoV-2, although their efficacy is still under evaluation [44,45]. Similarly, scientific research is focusing on the study of antiviral agents targeting specific viral genes and proteins in SARS-CoV-2, but such targets may not be feasible because their efficacy could be compromised by the rapid genetic mutations that CoVs undergo [46,47]. Prospects to overcome this problem and to optimize the use of antivirals should combine high-throughput screening, computational modeling, and structural biology, as well as the collaboration of different scientific sectors.

## 5. Conclusions

Although further research is needed to better clarify both the interaction between 6PP and BCoV proteins and the underlying mechanisms of its antiviral activity, these data demonstrated that prolonged 6PP treatment (72 h) significantly reduced BCoV viral load in cell monolayers. Moreover, antiviral activity was also observed when 6PP was administered following inhibition of viral internalization. The efficacy of 6PP in two different phases of BCoV infection may imply its ability to be used as a multi-stage antiviral, supporting its possible use in future translation research to evaluate the potential application of 6PP as an antiviral agent against SARS-CoV-2.

## Data Availability

The original contributions presented in this study are included in the article/Supplementary Material. Further inquiries can be directed to the corresponding authors.

## Supplementary Materials

The following supporting information can be downloaded at: https://www.mdpi.com/article/10.3390/vetsci12070634/s1, Figure S1: Multi-stage Fluorescent Image Transformation Protocol for all experimental and control samples at different conditions, with initial resizing of original images (A); following grayscale transformation (B) to optimize for computational efficiency and manipulation; and subsequently, HSV transformed for precise finetuning and hue channel parameterization (C); Figure S2: Computed fluorescence obtained by vectorization and aggregation of mean pixel intensity across all images; Table S1: Result of repeated-measures ANOVA on variables within and between treatment groups for immunofluorescence-positive samples; Table S2: Post hoc pairwise comparison; both P-unc and P-corr reported and where rm-ANOVA was significant; Table S3: Two-way ANOVA between the mean fluorescence in the control and the different experimental conditions.
