# Supplementary material for: In Vitro Antiviral Activity of the Fungal Metabolite 6-Pentyl-α-Pyrone Against Bovine Coronavirus: A Translational Study to SARS-CoV-2
Source: Vet Sci. 2025 Jul 2;12(7):634. doi: 10.3390/vetsci12070634 (PMC12298981; doi:10.3390/vetsci12070634)
Supplement: Supplementary file 1 [file vetsci-12-00634-s001.zip › vetsci-3686904-supplementary.pdf]

Supplementary material: Figures (Figures S1 and S2) and Tables (Tables S1-S3)

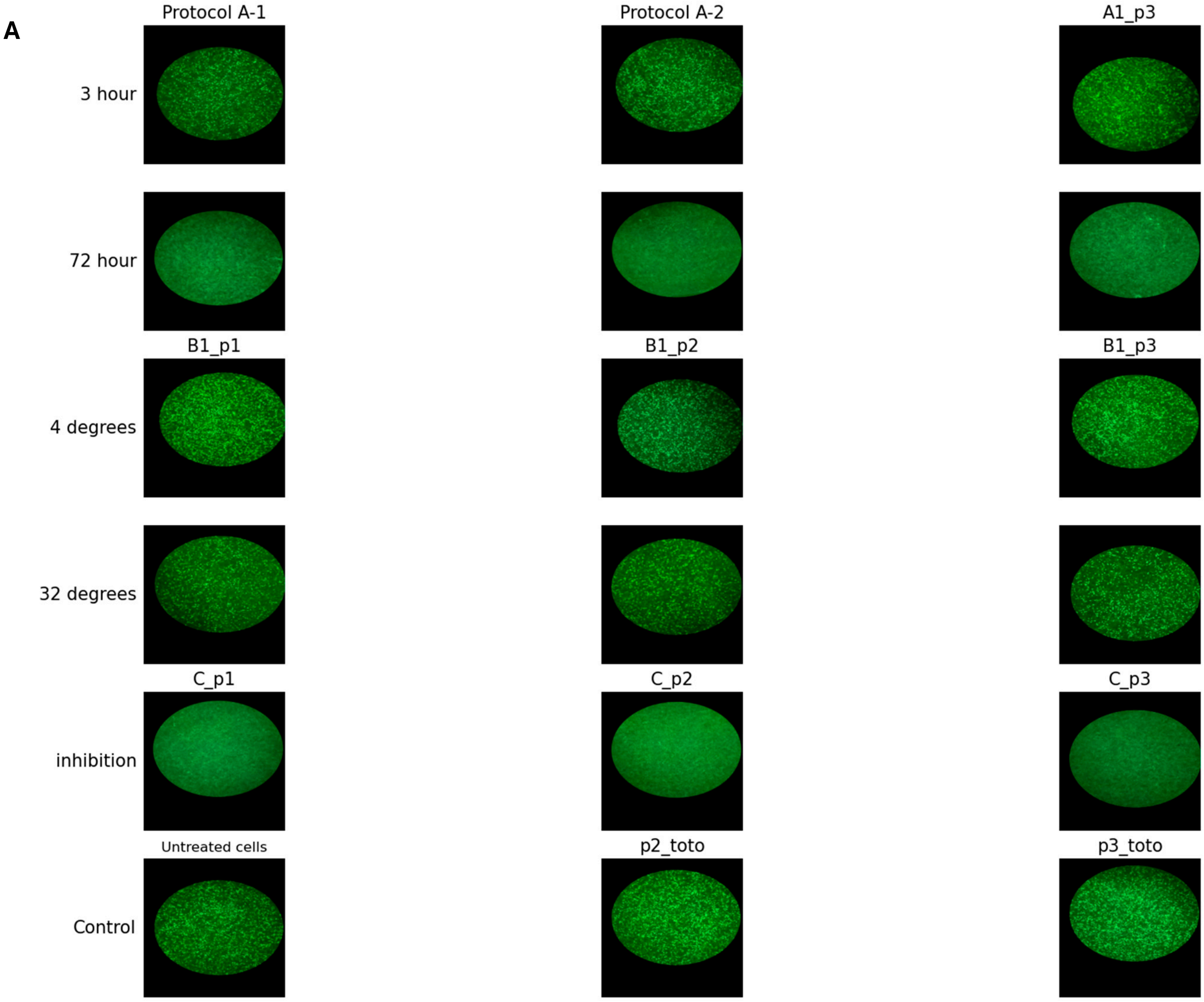

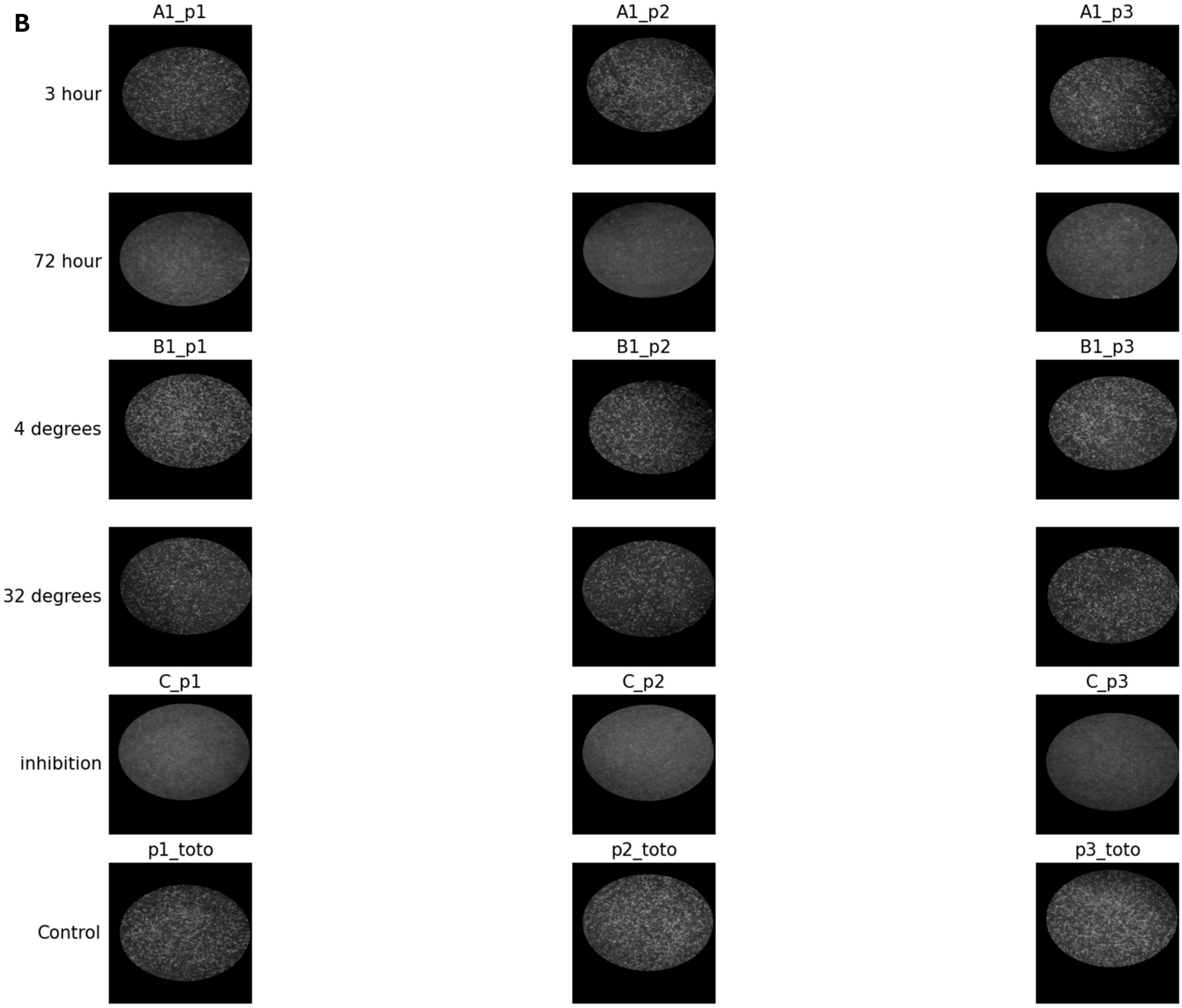
B
A1_p1
A1_p2
A1_p3
3 hour
72 hour
B1_p1
B1_p2
B1_p3
4 degrees
32 degrees
C_p1
C_p2
C_p3
inhibition
p1_toto
p2_toto
p3_toto
Control

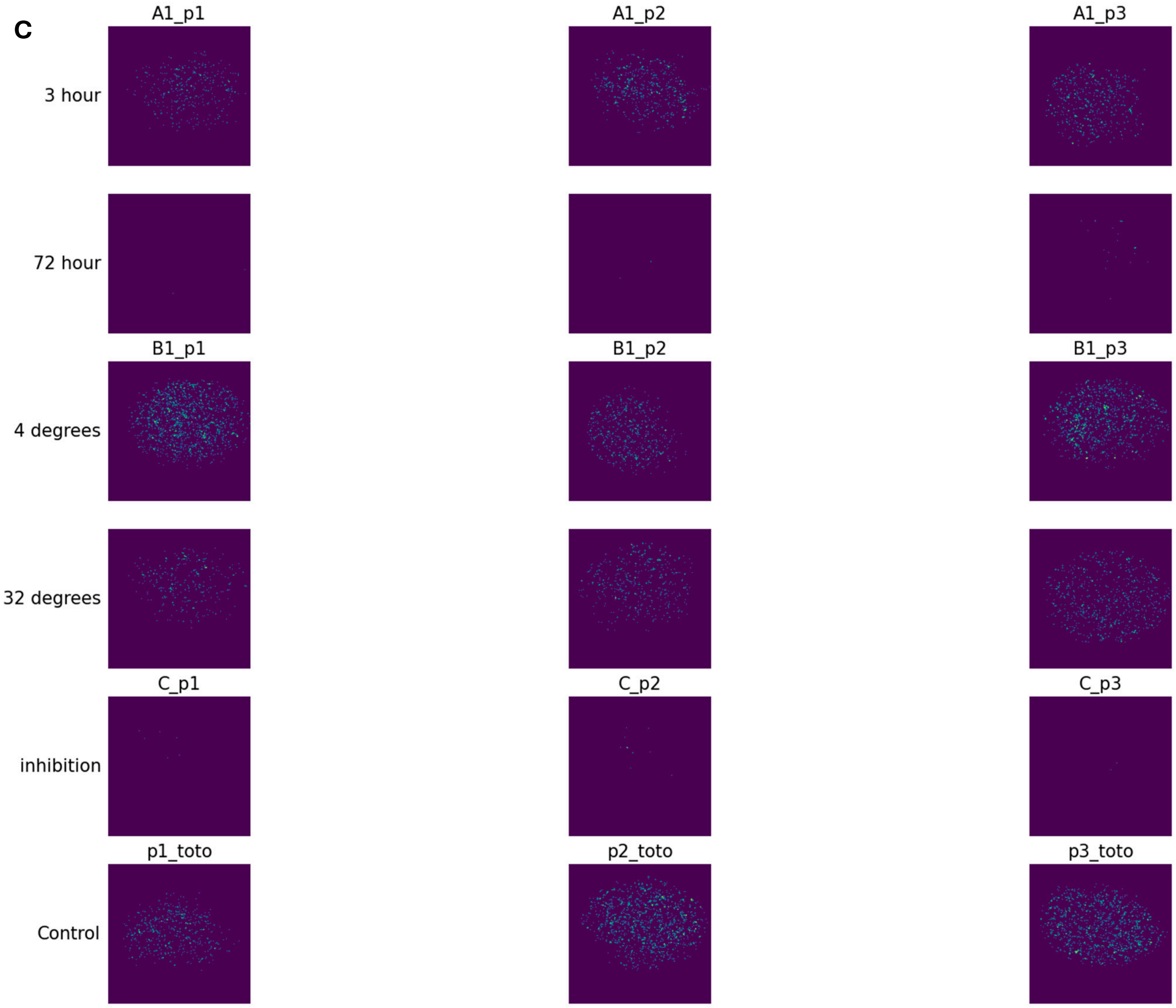

Figure. S1: Multi-stage Fluorescent Image Transformation Protocol for all experimental and control samples at different conditions, with initial resizing of original images (A); following grayscale transformation (B) to optimize for computational efficiency and manipulation; and subsequently, HSV transformed for precise finetuning and hue channel parameterization (C).

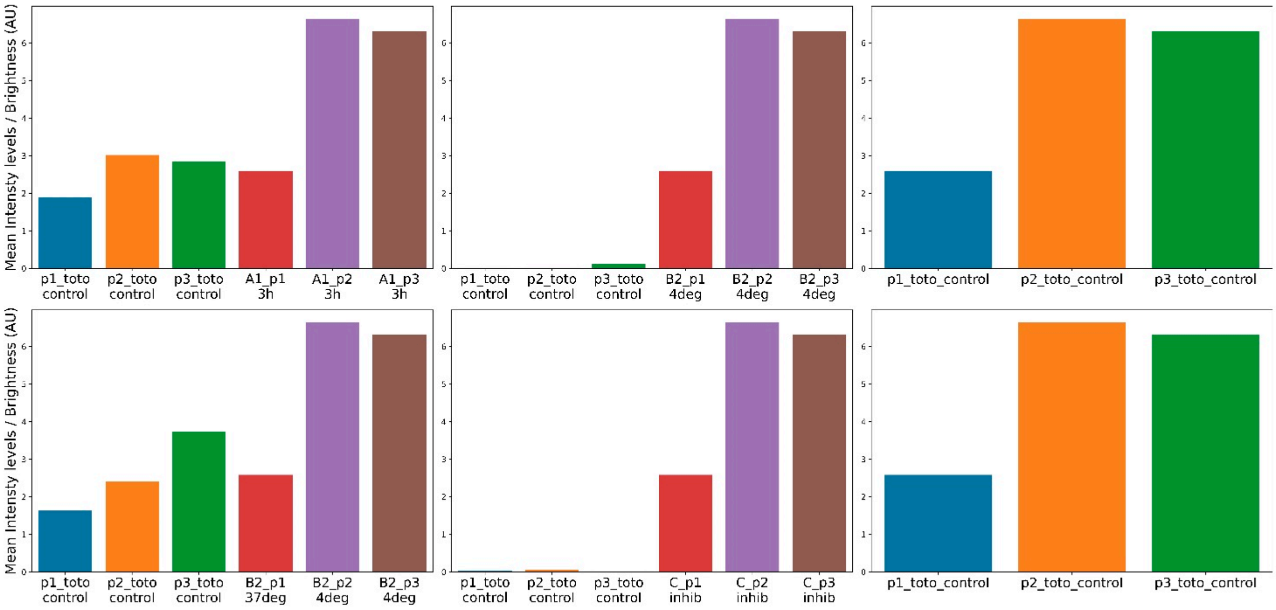

Figure. S2: Computed fluorescence obtained by vectorization and aggregation of mean pixel intensity across all images.

Table S1: Result of repeated-measures ANOVA on variables within and between treatment groups for immunofluorescence-positive samples.

**Category**	**Variable**		**Within subject**	**Df1**	**Df2**	**F-statistic**	***P*-unc**
	**Dependent**	**Explanatory**					
Virus-compound mixture (vc)	positives	time	temperature	2	6	0.429	0.670
	positives	time	temperature	2	4	0.182	0.840
Control virus mixture (cv)	positives	time	dilution	2	6	4.2	0.072
	positives	time	temperature	2	4	0.824	0.502
vc versus cv	positives	time	dilution	5	10	0.492	0.775
	positives	time	temperature	5	10	1.714	0.192

*Significant at $P \leq 0.05$; positives = samples positive for immunofluorescent test

Table S2: Post hoc pairwise comparison; both *P*-unc and *P*-corr reported and where rm-ANOVA was significant

**Category**	**Comparison**		**Bonferroni Corrected**	
	Variable 1	***Variable 2***	***P*-unc**	***P*-corr**
Virus-compound mixture	10^{-1}	10^{-3}	0.0153	0.0763
	10^{-3}	undiluted	0.0153	0.0763
Control virus mixture	10^{-3}	undiluted	0.0198	0.1188

Table S3: Two-way ANOVA between the mean fluorescence in the control and the different experimental conditions

	**Variable 1**	**Variable 2**	**Df**	**Mean_Sq**	**F**	***P*-value**
Protocol A	Control	A1 (3 hour)	1.0	10.10	3.699	0.127
		A1 (72 hour)	1.0	39.52	15.536	**0.0169***
Protocol B	Control	B1 (4 degree)	1.0	0.011	0.00235	0.964
		B1 (37 degree)	1.0	10.04	3.230	0.147
Protocol C	Control	C (Inhibition)	1.0	39.73	15.627	**0.0168***

*statistical significance at $P<0.05$
